# Correlations in Uniform Spanning Trees: a Fermionic Approach

**DOI:** 10.1007/s10955-025-03510-0

**Published:** 2025-10-18

**Authors:** Alan Rapoport

**Affiliations:** Utrecht University, Budapestlaan 6, 3584 CD Utrecht, The Netherlands

**Keywords:** Uniform spanning tree, Fermionic Gaussian free field, Scaling limit, Complete graph, Correlations, 60K35, 39A12, 60G15, 81V74

## Abstract

In the present paper we establish a clear correspondence between probabilities of certain edges belonging to a realization of the *uniform spanning tree* (UST), and the states of a *fermionic Gaussian free field*. Namely, we express the probabilities of given edges belonging or not to the UST in terms of fermionic Gaussian expectations. This allows us to explicitly calculate joint probability mass functions of the degree of the UST on a general finite graph, as well as obtain their scaling limits for certain regular lattices.

## Introduction

In Cipriani et al. [6] the authors study the joint moments of the so-called *height-one field* of the *Abelian sandpile model* (ASM), by means of a construction of a local field with *fermionic* variables on a graph. This was achieved given the fact that the height-one field of the ASM at stationarity can be put into correspondence with certain realizations of the *uniform spanning tree* (UST) [7, 8, 14]. By doing so, the authors also managed to obtain closed-form expressions of the joint moments of the degree field of the UST. In the present paper we build up on those techniques to obtain, among other results, a closed-form expression for the probability mass function of the UST.

Our first observation is a general recipe to calculate probabilities of given edges to be or not to be in the UST in terms of fermionic variables, which is the result given in Proposition 3.6 in Section 3. Namely, for any finite graph $\mathcal G = (\Lambda ,\,E)$ and directed edges $\{f_i\}_i$, $\{g_j\}_j$, for $\{f_i\}_i \cap \{g_j\}_j = \emptyset$, with tail points $\{v_i\}_i$, $\{w_j\}_j$ respectively,

1.1 $$\begin{aligned} \mathbb {P}\big (\{f_i\}_i \in \textrm{UST},\, \{g_j\}_j \notin \textrm{UST}\big ) = \left\langle \prod _{i}\nabla _{f_i}\psi (v_i) \nabla _{f_i}\overline{\psi }(v_i) \prod _{j}\left( 1- \nabla _{g_j}\psi (w_j) \nabla _{g_j}\overline{\psi }(w_j)\right) \right\rangle , \end{aligned}$$

where $\psi$ and $\overline{\psi }$ are generators of a Grassmannian algebra and $\left\langle \cdot \right\rangle$ is the so-called *fermionic Gaussian free field state* (fGFF) (roughly, expected values under the fGFF measure). Precise definitions will be given in Section 2, but for the moment we stress that these variables satisfy anti-commuting relations as

$$ \psi (v_i)\psi (v_j) = -\psi (v_j)\psi (v_i),\quad \forall \, i,\,j, $$

and the fGFF measure is a Gaussian measure on these variables.

On the other hand, there is a well-known connection between these UST probabilities and determinants of the *transfer-current matrix* *M*, which was originally studied to model electric networks. In this context, if $\mathcal G$ is considered as a network where each edge represents a conductance equal to 1, for any two edges *e* and *f* the value of $M(e,\,f)$ is the current measured through *f* when a battery imposes a unit current through *e*. These values can also be related to local times of a random walk on $\mathcal G$, so *M* can also be expressed in terms of gradients of the Green’s function of the graph in question (see e.g. Kassel and Wu [9]). With this ingredient, the aforementioned fermionic expected values can be written in terms of determinants of *M*.

At this point we should highlight that the connection between Grassmann variables and uniform spanning trees (USTs) is fundamentally explained by the Matrix Tree Theorem (MTT) (see for example  Bollobás [4]), which expresses the UST partition function as the determinant of a principal minor of the graph Laplacian. On the one hand, determinants arise naturally in the computation of Grassmannian Gaussian integrals, whereas the MTT provides the link between these determinants and the UST model. From another perspective, the nilpotency of Grassmann variables truncates any power expansion to first order, making them ideal for encoding high-temperature expansions in statistical mechanics. Although in the present paper we do not use Grassmann variables to compute new observables directly if not by means of the determinantal structure known by the MTT, they serve as a representation of observables of the UST in terms of a particular fermionic field. We leverage on the simplicity of formula (1.1) to encode observables of the UST in terms of straight-forward fermionic combinations. In Liu et al. [12], the authors look to find a conformal field theory (CFT) lying behind the UST. We hope that an enhancement of our techniques may serve to determine algebras and the defining such a CFT.

For $v\in \Lambda$ we can define the fields

$$ X_v^{(k_v)}{:=}\sum _{\mathcal {E}\subseteq E_v:\, |\mathcal {E}|=k_v} \prod _{e\in \mathcal {E}} \nabla _e\overline{\psi }(v) \nabla _e\psi (v) $$

for $k_v \in \{1,\,\ldots ,\,\deg _\mathcal {G}(v)\}$, being $\deg _\mathcal {G}(v)$ the degree of *v* on the graph $\mathcal G$, $E_v$ the edges incident to *v*, and

$$ Y_v{:=}\prod _{e\in E_v}\left( 1-\nabla _e\overline{\psi }(v) \nabla _e\psi (v)\right) . $$

With these fields we obtain the joint probability mass functions of the degree field $D_v$ of the UST as

$$ \mathbb {P}\left( D_v = k_v , \, v\in V\right) = \left\langle \prod _{v\in V} X_v^{(k_v)} Y_v\right\rangle . $$

This is the result of Theorem 3.7. We highlight that fermionic variables have already been used to study problems of random trees. See for example Caracciolo et al. [5] and Bauerschmidt et al. [3] for modern expositions on the subject and applications.

By means of the transfer-current matrix, this result can be further expanded to yield an explicit expression of the joint moments of the fields $\big (X_v^{(k_v)} Y_v\big )_v$ in terms of the Green’s function of the graph $\mathcal G$, given in Theorem 4.2. To the best of the author knowledge, in the literature there is no full general expression for the exact distribution of the degree field of a UST on a general graph. This can be applied, for example, to calculate the probability of a vertex on the complete graph $K_n$ to have degree *k*, for any $n\ge 1$. Taking $n\rightarrow \infty$, we show that the degree variable behaves as a Poisson variable plus 1, a result which was already known [2, 16], but it comes in a more straight-forward manner with our approach since we have an explicit expression of the probability mass function of the degree variable for any $n\ge 1$ at any given point.

Finally, if we take a bounded, open, connected subset $U\subset \mathbb {R}^d$ and restrict ourselves to a finite subset of regular lattices $\textbf{L}$ like $\mathbb {Z}^d$ or the triangular or hexagonal lattices in $d=2$ by taking the intersection $U/\varepsilon \cap \textbf{L}$ with $\varepsilon >0$, we can obtain a limiting expression for the joint cumulants of the variables $\big (X_v^{(k_v)} Y_v\big )_v$ when we take the limit of the whole infinite lattice, as

1.2 $$\begin{aligned} \tilde{\kappa }(v_1,\,\dots ,\,v_n)&{:=}\lim _{\varepsilon \rightarrow 0} \varepsilon ^{-dn} \kappa \left( \left( X_v^{k_v}\right) ^\varepsilon \, Y_v^\varepsilon :\,v\in V\right) \nonumber \\&= - \left[ \prod _{v\in V}C_\textbf{L}^{(k_v)}\right] \sum _{\sigma } \sum _{\eta } \prod _{v\in V} \partial _{\eta (v)}^{(1)}\partial _{\eta (\sigma (v))}^{(2)} g_U\left( v,\, \sigma (v)\right) , \end{aligned}$$

where $g_U$ is the continuum Green’s function on *U*, $\sigma$ are cyclic permutations on *V*, and $\eta$ are the directions of derivation on $\mathbb {R}^d$. Once again, the notation will become more clear after Section 2. The constants $C_\textbf{L}^{(k_v)}$ are explicitly calculated in terms of the Green’s function values of $\textbf{L}$. We observe that the expression for the limiting cumulants are the same for all lattices up to a constant, hinting towards a potential universality property of the system. Unlike in  Cipriani et al. [6], the proof of this now more general limiting result is unified for all the lattices considered, which makes the necessary conditions of the lattice more clear for our proof to work. The reader will also observe that expression (1.2) has exactly the same functional form as that of the height-one field of the ASM [6, 7], albeit with a different constant in front, meaning that the limiting joint cumulants expressions are affected by the values of $(k_v)_v$ only through $C_{\textbf{L}}^{(k_v)}$, but otherwise remain the same.

**Structure of the paper.** We begin our paper setting up notation and defining the main objects of interest in Section 2. Section 3 is devoted to recapitulate on the fermionic formalism used throughout the paper, as well as stating the first general results linking fermionic Gaussian states and UST probabilities. The moments/cumulants, both for a finite graph and the limiting case, are in Section 4. At the end of that section we also discuss the case of the complete graph $K_n$ and its limit $n\rightarrow \infty$. Finally, Section 5 is devoted to the proofs of the main theorems.

## Notation and Definitions

**Lattices, sets and functions** For the rest of the paper *d* will denote the dimension of the underlying space we work on. We will write |*A*| for the cardinality of a set *A*. For $n\in \mathbb {N}$, let [*n*] denote the set $\{1,\,\ldots ,\,n\}$.

Throughout the paper $\textbf{L}$ will denote a lattice. In particular, we will consider what we will call the hypercubic lattice $\mathbb {Z}^d$, the two dimensional triangular lattice $\textbf{T}$, and we will also make some remarks on the two dimensional hexagonal lattice $\textbf{H}$. Since these lattices are regular, we write $\deg _{\textbf{L}}$ for the degree of any vertex, which is 2*d* for $\mathbb {Z}^d$, 6 for $\textbf{T}$, and 3 for $\textbf{H}$.

We will denote an oriented edge *f* on the lattice $\textbf{L}$ as the ordered pair $(f^-,\,f^+)$, being $f^-$ the tail and $f^+$ the tip of the edge. Denote $\{e_i\}_{i\in [\deg _{\textbf{L}}]}$ the set of edges with tail in the origin. The $e_i$’s define a natural orientation of edges which we will tacitly choose whenever we need oriented edges (for example when defining the matrix *M* in (2.5)). The opposite vectors will be written as $e_{d+i} {:=}-e_i,\,i=1,\,\ldots ,\,d$. Furthermore

$$ \tilde{e}_i {:=}(0,\,\ldots ,\,0,\,\underbrace{1}_{i\text {-th position}},\,0,\,\ldots ,\,0),\quad i=1,\,\ldots ,\,d $$

denotes the *d* standard coordinate vectors of $\mathbb {R}^d$.

The collection of all $e_i$, $i\in \{1,\,\ldots ,\,\deg _{\textbf{L}}\}$, will be called $E_o$, where *o* is the origin. By abuse of notation but convenient for the paper, if $f=(f^-,\,f^-+e_i)$ for some $i\in \left[ \deg _{\textbf{L}}\right]$, we denote by $-f$ the edge $(f^-,\,f^- - e_i)$ whenever it exists; that is, the reflection of *f* over $f^-$.

Let $A \subseteq \mathbb {R}^d$ be a countable set. For every $v \in A$, denote by $E_v$ the set $E_o+v$, and let $E(A) = \bigcup _{v\in A} E_v$.

Let $U\subseteq \mathbb {R}^d$ and $e \in E_o$. For a function $g:\,U\rightarrow \mathbb {R}^d$ differentiable at *x* we define $\partial _e g(x)$ as the directional derivative of *g* at *x* in the direction corresponding to *e*, that is

$$\begin{aligned} \partial _e g(x) = \lim _{t \rightarrow 0^{+}} \frac{g(x+t e)-g(x)}{t}. \end{aligned}$$

Likewise, when we consider a function in two variables $g: \mathbb {R}^d \times \mathbb {R}^d \rightarrow \mathbb {R}$, we write then $\partial ^{(j)}_e g(\cdot ,\cdot )$ to denote the directional derivative in the *j*-th entry, $j=1,\,2$.

**Graphs and Green’s function** As we use the notation $(u,\,v)$ for a directed edge we will use $\{ u,\, v\}$ for the corresponding undirected edge. For a finite (unless stated otherwise) graph $\mathcal {G}=(\Lambda ,\, E)$ we denote the degree of a vertex *v* by $\deg _\mathcal {G}(v) {:=}\left| \{u \in \Lambda :\, u \sim v\}\right|$, where $u\sim v$ means that *u* and *v* are nearest neighbors.

### Definition 2.1

(*Discrete derivatives)* For a function $g:\,\textbf{L}\rightarrow \mathbb {R}$, its discrete derivative $\nabla _{e_i} g$ in the direction $i=1,\,\ldots ,\,\deg _{\textbf{L}}$ is defined as

$$ \nabla _{e_i} g(u){:=}g(u+e_i)-g(u),\quad u\in \textbf{L}. $$

Analogously, for a function $g:\,\textbf{L}\times \textbf{L}\rightarrow \mathbb {R}$ we use the notation $\nabla ^{(1)}_{e_i}\nabla ^{(2)}_{e_j} g$ to denote the double discrete derivative defined as

$$ \nabla ^{(1)}_{e_i}\nabla ^{(2)}_{e_j}g(u,\,v){:=}g(u+e_i,\,v+e_j)- g(u+e_i,\,v)-g(u,\,v+e_j)+g(u,\,v), $$

for $$u,\,v\in \textbf{L}$$, $$i,\,j=1,\,\ldots ,\,\deg _{\textbf{L}}$$.

### Definition 2.2

(*Discrete Laplacian on a graph)* We define the (unnormalized) *discrete Laplacian* on $\textbf{L}$ as2.1where $u,\,v \in \textbf{L}$ and $u \sim v$ denotes that *u* and *v* are nearest neighbors. For any function $g:\,\textbf{L}\rightarrow \mathcal A$, where $\mathcal A$ is an algebra over $\mathbb {R}$, we define

2.2 $$\begin{aligned} \Delta g(u) {:=}\sum _{v \in \textbf{L}}\Delta (u,\,v) g(v) = \sum _{v \sim {u}} (g(v)-g(u)). \end{aligned}$$

Note that we define the function taking values in an algebra because we will apply the Laplacian both on real-valued functions and functions defined on Grassmannian algebras, which will be introduced in Section 3.

We also introduce $\Delta _\Lambda {:=}(\Delta (u,\,v))_{u,\,v\in \Lambda }$, the restriction of $\Delta$ to $\Lambda$. Notice that for any lattice function *f* we have that for all $u \in \Lambda$,

2.3 $$\begin{aligned} \Delta _\Lambda g(u) = \sum _{v \in \Lambda } \Delta (u,v) g(v) = \Delta g_\Lambda (u) \end{aligned}$$

where $g_\Lambda$ is the lattice function given by .

The exterior boundary of a set $\Lambda$ will be defined by

$$ \partial ^{\textrm{ex}}\Lambda {:=}\{u\in \textbf{L}\setminus \Lambda :\,\exists \,v\in \Lambda :\,u\sim v\}. $$

### Definition 2.3

(*Discrete Green’s function*) Let $u \in \Lambda$ be fixed. The Green’s function $G_\Lambda (u,\cdot )$ with Dirichlet boundary conditions is defined as the solution ofwhere $\Delta _\Lambda$ is defined in (2.3).

### Definition 2.4

(*Infinite volume Green’s function*, [11, Sec. 1.5-1.6]) With a slight abuse of notation we denote by $G_0(\cdot ,\cdot )$ two objects in different dimensions:

- $d\ge 3$ (only for $\mathbb {Z}^d$): $G_0$ is the solution of
- $d=2$: $G_0$ is given by
  $$\begin{aligned} G_0(u,\,v)= -\frac{1}{\deg _{\textbf{L}}(u-v)}a(u-v),\quad u,\,v\in \textbf{L}, \end{aligned}$$
  where $a(\cdot )$ is the potential kernel defined as
  $$ a(u) = \sum _{n=0}^\infty \big [\mathbb {P}_o(S_n=o)-\mathbb {P}_o(S_n=u)\big ], \quad u\in \textbf{L}, $$
  and $\{S_n\}_{n\ge 0}$ is a random walk on the plane starting at the origin and $\mathbb {P}_o$ its probability measure.

**Cumulants** We now give a brief recap of the definition of cumulants and joint cumulants for random variables. Let $n\in \mathbb {N}$ and $\textbf{X}=(X_{i})_{i=1}^n$ be a vector of real-valued random variables, each of which has all finite moments.

### Definition 2.5

(*Joint cumulants of random vector*) The cumulant generating function $K(\textbf{t})$ of $\textbf{X}$ for $\textbf{t}=(t_1,\,\dots ,\,t_n)\in \mathbb {R}^n$ is defined as

$$ K(\textbf{t}) {:=}\log \left( \mathbb {E}\big [e^{ \textbf{t}\cdot \textbf{X}}\big ]\right) = \sum _{\textbf{m}\in \mathbb {N}^n} \kappa _{\textbf{m}}(\textbf{X}) \prod _{j=1}^n \frac{t_j^{m_j}}{m_j!} \ , $$

where $\textbf{t}\cdot \textbf{X}$ denotes the scalar product in $\mathbb {R}^n$, $\textbf{m}=(m_1,\,\dots ,\,m_n)\in \mathbb {N}^n$ is a multi-index with *n* components, and

$$ \kappa _{\textbf{m}}(\textbf{X})=\frac{\partial ^{|m|}}{\partial t_1^{m_1}\cdots \partial t_n^{m_n}}K(\textbf{t})\Big |_{t_1=\ldots =t_n=0} \ , $$

being $|m|=m_1+\cdots +m_n$. The joint cumulant of the components of $\textbf{X}$ can be defined as a Taylor coefficient of $K(t_1,\,\ldots ,\,t_n)$ for $\textbf{m}=(1,\,\ldots ,\,1)$; in other words

$$ \kappa (X_1,\,\ldots ,\,X_n)=\frac{\partial ^n}{\partial t_1\cdots \partial t_n} K(\textbf{t})\Big |_{t_1=\ldots =t_n=0} \ . $$

In particular, for any $A\subseteq [n]$, the joint cumulant $\kappa (X_i:\, i\in A)$ of $\textbf{X}$ can be computed as

$$ \kappa (X_i:\, i\in A) = \sum _{\pi \in \Pi (A)} (|\pi |-1)! (-1)^{|\pi |-1} \prod _{B\in \pi } \mathbb {E} \left[ \prod _{i\in B} X_i \right] \ , $$

with $|\pi |$ the cardinality of $\pi$.

Let us remark that, by some straightforward combinatorics, it follows from the previous definition that

2.4 $$\begin{aligned} \mathbb {E} \left[ \prod _{i\in A} X_i \right] = \sum _{\pi \in \Pi (A)} \prod _{B\in \pi } \kappa (X_i:\, i\in B) \ . \end{aligned}$$

If $A=\{i,\,j\}$, $i,\,j\in [n]$, then the joint cumulant $\kappa (X_i,\,X_j)$ is the covariance between $X_{i}$ and $X_{j}$. We stress that, for a real-valued random variable *X*, one has the equality

$$ \kappa (\underbrace{X,\,\ldots ,\,X}_{n\text { times}})=\kappa _n(X),\quad n\in \mathbb {N}, $$

which we call the *n-th cumulant of X*.

**Good sets and transfer-current matrix** We need to introduce a technical requirement for the sets we will study in the theorems that follow, that prevents us from choosing points that share edges. This requirement can however be circumvented, as we show in Section 4.

### Definition 2.6

(*Good set*) We call $A \subseteq \Lambda$ a *good set* if it does not contain any nearest neighbors. That is, $||v-u|| > 1$ for any $u,\,v\in A$.

Finally, we need the notion of the *transfer-current matrix*, a key ingredient in many expressions we obtain in our theorems.

### Definition 2.7

(*Transfer-current matrix*) We define the *transfer-current matrix* $M_\Lambda$ as

2.5 $$\begin{aligned} M_\Lambda (f,\,g) {:=}\nabla _{\eta ^*(f)}^{(1)}\nabla _{\eta ^*(g)}^{(2)}G_{\Lambda }(f^{-},\,g^-) ,\quad f,\,g \in E(\Lambda ), \end{aligned}$$

where $\eta ^*(f)\in E_o$ is the coordinate direction induced by $f\in E(\Lambda )$ on $f^-$ (in the sense that $\eta ^*(f)=e_i$ if $f=(f^-,\, f^-+e_i)$). Hereafter, to simplify notation we will omit the dependence of $M_\Lambda$ on $\Lambda$ and simply write *M*.

### Remark 1

As stated in Lyons and Peres [13], there is another interpretation of *M* in terms of electrical networks, as follows: let $\mathcal G$ represent an electric network with impedance 1 on each edge. Defining $\phi _{f}(x)$ as the voltage at vertex $x\in \Lambda$ when a battery of 1 volt is connected between vertices $g^-$ and $g^+$ by removing the resistance on *g* and setting the voltage at $f^-$ to 0, $M(f,\,g)$ is given by

$$ M = \phi _f(g^+) - \phi _f(g^-) . $$

## Fermionic Formalism

As we will see in this section, working with Grassmann variables (i.e., fermions) allow us to express properties from the UST. We will not give a complete exposition of the subject in this paper; however, the interested reader can resort to Cipriani et al. [6] for a similar setting to the one used here, or  Abdesselam [1], Meyer [15] for a more comprehensive presentation.

### Definition 3.1

(Abdesselam [1, Definition 1]) Let $M\in \mathbb {N}$ and $\xi _1,\,\ldots ,\,\xi _M$ be a collection of letters. Let $\mathbb {R}\left[ \xi _{1},\,\ldots ,\,\xi _{M}\right]$ be the quotient of the free non-commutative algebra $\mathbb {R}\langle \xi _1,\,\ldots ,\,\xi _M\rangle$ by the two-sided ideal generated by the anticommutation relations

3.1 $$\begin{aligned} \xi _j \xi _j = - \xi _i \xi _j, \end{aligned}$$

where $i,\,j \in [M]$. We will denote it by $\Omega ^{M}$ and call it the Grassmann algebra in *M* variables. The $\xi$’s will be referred to as *Grassmannian variables* or *generators*. Due to anticommutation these variables are called “fermionic” (as opposed to commutative or “bosonic” variables).

Notice that, due to the anticommutative property, we have that for any variable Pauli’s exclusion principle holds [1, Proposition 2]:

3.2 $$\begin{aligned} \xi _i^2 = 0, \quad i\in [M]. \end{aligned}$$

### Definition 3.2

(*Grassmannian–Berezin integration*) The Grassmann–Berezin integral is defined as

$$ \int F d \xi {:=}\partial _{\xi _{M}}\partial _{\xi _{M-1}} \cdots \partial _{\xi _2}\partial _{\xi _1}F, \quad F\in \Omega ^{M}. $$

On the grounds of this definition, for the rest of the paper Grassmannian–Berezin integrals will be denoted by $\left( \prod _{i=1}^M\partial _{\xi _i}\right) F$.

The most important result that we will use in this work is as follows. For a given matrix $A = (A_{i,\,j})_{i \in I_0,\, j \in J_0}$, and $I \subseteq I_0,\, J\subseteq J_0$, such that $|I|=|J|$, we write $\det (A)_{IJ}$ to denote the determinant of the submatrix $(A_{i,\,j})_{i \in I,\, j \in J}$. When $I=J$, we simply write $\det (A)_I$.

### Theorem 3.3

(Wick’s theorem for “complex” fermions) Let *A* be an ${m\times m}$, *B* an ${r\times m}$ and *C* an ${m\times r}$ matrix respectively with coefficients in $\mathbb {R}$. For any sequences of indices $I = \{i_1,\,\dots ,\,i_r\}$ and $J = \{j_1,\,\dots ,\,j_r\}$ in [*m*] of the same length *r*, if the matrix *A* is invertible we have

1. $$\displaystyle \left( \prod _{i=1}^m\partial _{\overline{\psi }_i}\partial _{\psi _i}\right) \prod _{\alpha =1}^r \psi _{i_\alpha }\overline{\psi }_{j_\alpha }\exp \left( (\varvec{\psi },\,A\varvec{\overline{\psi }})\right) = \det (A) \det \left( A^{-\intercal }\right) _{IJ}$$,
2. $$\displaystyle \left( \prod _{i=1}^m\partial _{\overline{\psi }_i}\partial _{\psi _i}\right) \prod _{\alpha =1}^r (\psi ^T C)_\alpha (B\overline{\psi })_\alpha \exp \left( (\varvec{\psi },\,A\varvec{\overline{\psi }})\right) = \det (A) \det \left( BA^{-1}C\right) $$.

If $|I| \ne |J|$, the integral is 0.

### Definition 3.4

(*Fermionic Gaussian free field*) The normalized fermionic Gaussian free field state is the linear map $\left\langle \cdot \right\rangle :\, \Omega ^{2\Lambda } \rightarrow \mathbb {R}$ defined as

$$ \left\langle F\right\rangle {:=}\frac{1}{\det (-\Delta _\Lambda )} \left( \prod _{v\in \Lambda }\partial _{\overline{\psi }_v}\partial _{\psi _v}\right) \exp \left( \langle \varvec{\psi },\,-\Delta _\Lambda \varvec{\overline{\psi }}\rangle \right) F,\quad F\in \Omega ^{2\Lambda }. $$

### Fermions and the Uniform Spanning Tree

Now that we have the key ingredients to work with fermions, we will see how these objects allow us to study probabilistic behaviors of the edges of a realization of the UST measure.

We will first consider gradients of the generators in the following sense:

#### Definition 3.5

(*Gradient of the generators*) The gradient of the generators in the *i*-th direction is given by

$$ \nabla _{e_i}\psi (v)=\psi _{v+e_i}-\psi _v,\quad \nabla _{e_i}\overline{\psi }(v)=\overline{\psi }_{v+e_i}-\overline{\psi }_v,\quad v\in \Lambda ,\,i=1,\,\ldots ,\,\deg _\mathcal {G}(v). $$

Define $$\zeta (e)$$ as

$$ \zeta (e) {:=}\nabla _e\psi (e^-)\nabla _e\overline{\psi }(e^+), $$

and observe that the elements $\zeta (\cdot )$ are commutative, that is,

$$ \zeta (a)\zeta (b) = \zeta (b)\zeta (a),\quad \forall \, a,\,b \in E, $$

but we still have that $\zeta (a)^2 = 0$. These objects are key when analyzing probabilities of edges showing up in the UST, in the sense of the result that follows.

#### Proposition 3.6

Let the tree *T* be a realization of the UST measure. For $F,\,G \subseteq E$, $F\cap G = \emptyset$ it holds that

$$ \mathbb {P}\left( F \subseteq T,\, G\cap T = \emptyset \right) = \left\langle \prod _{f\in F} \zeta (f) \prod _{g\in G} \left( 1 - \zeta (g)\right) \right\rangle = \det \big (M^{(|F|)}\big ), $$

where

#### Proof

Observe that

$$ \prod _{g\in G}(1-\zeta (g)) = \sum _{\gamma \subseteq G}(-1)^{|\gamma |}\prod _{g\in \gamma }\zeta (g), $$

so that

$$\begin{aligned} \left\langle \prod _{f\in F} \zeta (f) \prod _{g\in G}(1-\zeta (g))\right\rangle= &   \sum _{\gamma \subseteq G}(-1)^{|\gamma |} \left\langle \prod _{g\in \gamma }\zeta (g) \prod _{f\in F}\zeta (f)\right\rangle \\= &   \sum _{\gamma \subseteq G} (-1)^{|\gamma |} \,\mathbb {P}\left( F \subseteq T,\,\gamma \subseteq T\right) . \end{aligned}$$

Using the inclusion-exclusion principle, we obtain the first equality. The equality between the first and third members follows from Pemantle [16, Theorem 4.3] (noting that there is a typo in their definition of $M^{(|F|)}$). $\square$

#### Remark 2

In view of Proposition 3.6 we have the following recipe to cook up a field whose expectation matches that of the UST: for each edge *f* we want in the UST, add a factor $\zeta (f)$, and for each edge *g* we do not want, add a factor $1-\zeta (g)$. Observe that, once we add an edge *e* by adding the factor $\zeta (e)$, then adding another factor $1-\zeta (e)$ does nothing. This is easily seen from the fact that

$$ \zeta (e) \left( 1-\zeta (e)\right) = \zeta (e) - \zeta (e)^2 = \zeta (e) . $$

### Degree of the Uniform Spanning Tree

So far we have seen the relationships between fermionic variables and particular edges on a spanning tree. We will now use those results to study the behaviour of the degree of a realization of the UST measure at given points on the graph.

#### Remark 3

Until now we have defined edges on graphs to be oriented. However, in the following definitions the orientation play no rôle, so we will consider edges as non oriented.

Let $\mathcal G = (\Lambda ,\,E)$ be any graph. For each $v\in \Lambda$ and $k_v \in \{1,\,\dots ,\,\deg _{\mathcal {G}}(v)\}$, we define the field $\mathbf {X^{(k)}} = \big (X_v^{(k_v)}\big )_{v\in V}$ as

3.3 $$\begin{aligned} X_v^{(k_v)}{:=}\sum _{\mathcal {E}\subseteq E_v:\, |\mathcal {E}|=k_v} \prod _{e\in \mathcal {E}} \zeta (e),\quad v\in \Lambda . \end{aligned}$$

In view of Remark 2, this is equivalent to asking that the degree of the UST at a point *v* is at least $k_v$; that is,If $k_v=1$ for all *v*, this is just the field $(X_v)_v$ defined in Cipriani et al. [6]. Observe also that, because of the nilpotency property of fermions,

$$ X^{(k_v)}_v = (X_v)^{k_v}, $$

so we will sometimes indistinctly denote it as $X_v^{k_v}$ The same applies for $\textbf{X}^{(\textbf{k})}$ written as $\mathbf {X^k}$. We will also need auxiliary Grassmannian observables $\textbf{Y}=(Y_v)_{v\in V}$ given by

3.4 $$\begin{aligned} Y_v{:=}\prod _{e\in E_v}\left( 1-\zeta (e)\right) ,\quad v\in \Lambda . \end{aligned}$$

Define the degree field of the UST $(D_v)_{v\in \Lambda }$ as3.5which is “equal” (in the sense of its finite-dimensional distributions) to $\big (X_v\big )_v$, as it was seen in Cipriani et al. [6]. More precisely, for $V\subseteq \Lambda$ a good set (neighboring points will be dealt with in Section 4),

$$ \mathbb {E}\left[ \prod _{v\in V}D_v\right] = \left\langle \prod _{v\in V}X_v\right\rangle . $$

For $k_v\in \{1,\,\dots ,\,\deg _\mathcal {G}(v)\}$, define the degree-$k_v$ field asAs a consequence of Proposition 3.6, we can express the probability of the degree of the UST at different non-neighboring points as follows:

#### Theorem 3.7

Let $V\subset \Lambda$ be a good set. For any $k_v\in \{1,\,\dots ,\,\deg _\mathcal {G}(v)\}$, with $v\in V$, it holds that

3.6 $$\begin{aligned} \mathbb {P}\left( D_v = k_v , \, v\in V\right) = \mathbb {E}\left[ \prod _{v\in V}\delta ^{(k_v)}_v\right] = \left\langle \prod _{v\in V} X_v^{k_v} Y_v\right\rangle . \end{aligned}$$

Note that this is a generalization of Cipriani et al. [6, Theorem 3.1], where we obtain the same result for $k_v=1$ for all $v\in V$, even though in that case our main focus was the height-one field of the Abelian sandpile model.

#### Remark 4

Observe that points in *V* need to be different. In fact, for $v\in V$,

$$ \mathbb {E}\left[ D_v^2\right] \ne \left\langle X_v^2\right\rangle , $$

and of course neither does it hold for larger powers. This is because the square of an indicator function (see (3.5)) is the same indicator, whereas the square of $\zeta (e)$, $e\in E$ (see (3.3)), is 0. However, using Proposition 3.6 we observe that

$$ \left\langle X_v(X_v+1)\right\rangle = \left\langle X_v^2\right\rangle + \left\langle X_v\right\rangle = \sum _{\begin{array}{c} e,\,f\in E_v\\ e\ne f \end{array}}\det {(M)}_{e,\,f} + \sum _{e\in E_v} M(e,\,e) = \mathbb {E}\left[ D_v^2\right] . $$

Following the same reasoning,

$$ \mathbb {E}\big [D_v^m\big ] = \sum _{i\in [m]} a_i \left\langle X_v^i\right\rangle , $$

where the coefficients $a_i$ correspond to a modification of the binomial coefficients. More precisely, for

$$ a_i = \left( {\begin{array}{c}m\\ i-1\end{array}}\right) , $$

while for

$$ a_i = \left( {\begin{array}{c}m\\ i\end{array}}\right) . $$

We could also find the reverse expression, that is, $\left\langle X_v^m\right\rangle$ as a function of $\mathbb {E}[D_v^i]$, $i\in [m]$. We can use the results on Pemantle [16, Section 5.2] to obtain

$$ \left\langle X_v^m\right\rangle = m!\, \mathbb {E}\left[ \left( {\begin{array}{c}D_v\\ m\end{array}}\right) \right] = \mathbb {E}\left[ \prod _{i=0}^{m-1}(D_v-i)\right] $$

for any $$m\in \mathbb {N}$$.

## Cumulants of the UST Degree

We will now study the cumulants (related to moments, as seen in Section 2) of the fields $\mathbf{X^k \, Y}$ on an arbitrary graph, and then obtain limiting expressions for some particular lattices. The next theorem is a generalization of Cipriani et al. [6, Theorem 3.5] when $k_v=1$ for all points $v\in V$. Before that, let us define some key elements for our theorems.

**General definitions.** Let $\Lambda$ be a finite and connected (in the usual graph sense) subset of a lattice $\textbf{L}$ and $V \subseteq \Lambda$ be a good set according to Definition 2.6. As *V* is a good set, notice that every edge in *E*(*V*) is connected to exactly one vertex in *V*.

For any finite set *A* we denote the set of permutations of *A* by *S*(*A*). Furthermore, we write $S_{{{\,\textrm{cycl}\,}}}(A)$ to denote the set of *cyclic* permutations of *A* (without fixed points).

**Permutations: connected and bare.** We define the multigraph $V_\tau = \left( V,\, E_\tau (V)\right)$ induced by $\tau$ in the following way. For each pair of vertices $v \ne w$ in *V*, we add one edge between *v* and *w* for each $f \in E_v, f' \in E_w$ such that either $\tau (f)= f'$ or $\tau (f')= f$. If $v=w$, we add no edge, so $\deg _{V_\tau }(v) \le |E_v|$.

Fix $A\subseteq E(V)$ such that $E_v \cap A \ne \emptyset$ for all $v \in V$, i.e. we have a set of edges with at least one edge per vertex of *V*. Let $\tau \in S(A)$ be a permutation of edges in *A*.

### Definition 4.1

(*Connected and bare permutations*) Let $\Lambda \subseteq \textbf{L}$ finite, *V* good as in Definition 2.6, $|V|\ge 2$, $A\subseteq E(V)$ and $\tau \in S(A)$ be given.

- We say that $\tau$ is *connected* if the multigraph $V_\tau$ is a connected multigraph.
- We say that $\tau$ is *bare* if it is connected and $\deg _{V_\tau }(v)=2$ for all $v\in V$ (it is immediate to see that the latter condition can be replaced by $|E_\tau (V)|=|V|$).

If $|V|=1$, as it can happen in Theorem 4.2, we consider every permutation $\tau \in S(A)$ as both connected and bare.

**Fig. 1:**
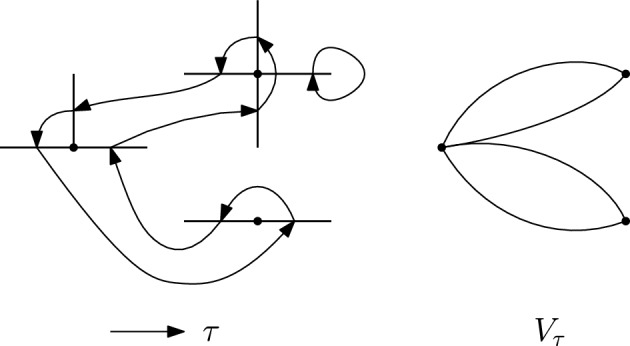
A connected permutation $\tau$ on edges of $\mathbb {Z}^2$ and the multigraph $V_\tau$ associated to it. Notice that this permutation is **not** bare

**Fig. 2:**
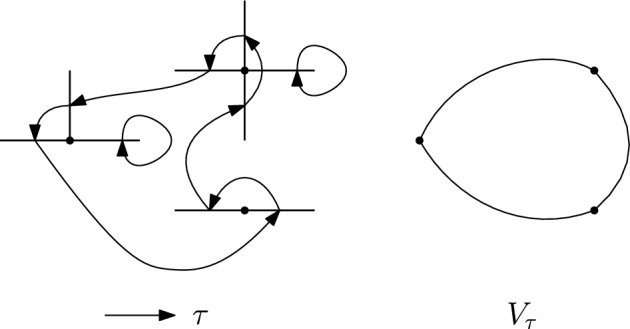
A bare permutation $\tau$ on edges of $\mathbb {Z}^2$ and the multigraph $V_\tau$ associated to it

We will denote by $S_{{{\,\textrm{co}\,}}}(A)$ the set of connected permutations in *S*(*A*), and by $S_{{{\,\textrm{bare}\,}}}(A)$ the set of bare permutations. See Figures 1 and 2 for some examples, where the mapping $\tau (f)=f'$ is represented via an arrow $f\rightarrow f'.$

**Fig. 3:**
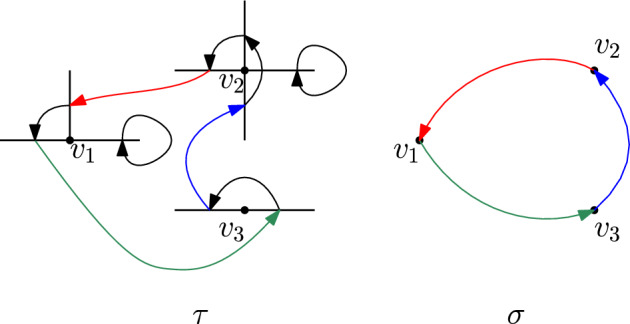
A bare permutation $\tau$ on edges and the induced permutation $\sigma$ on points, in $d=2$

For $\tau$ bare we have, by definition, that for each *v* there are exactly two edges $f,\,f^\prime \in A$ (possibly the same) such that $\tau (f') \not \in E_v$ and $\tau ^{-1} (f) \not \in E_v$. We will refer to this as $\tau$ enters *v* through *f* and exits *v* through $f^\prime$. Therefore, for any bare permutation $\tau \in S_{{{\,\textrm{bare}\,}}}(A)$, we can define an induced permutation on vertices $\sigma = \sigma _\tau \in S_{{{\,\textrm{cycl}\,}}}(V)$ given by $\sigma (v)=w$ if there there exists (a unique) $f \in E_v$ and $f' \in E_w$ such that $\tau (f)=f'$. Figure 3 shows an example in $\mathbb {Z}^2$.

We are now ready to state the theorems. Let *U* be a bounded, open, connected subset of $\mathbb {R}^d$ with smooth boundary, and define $U_\varepsilon {:=}U/\varepsilon \cap \textbf{L}$ for $\varepsilon >0$. For any $v\in U$, let $v_\varepsilon$ be the discrete approximation of *v* in $U_\varepsilon$; that is, . Define $g_U$ as the continuum harmonic Green’s function on *U* with 0-boundary conditions outside *U*. We write $X_v^\varepsilon$, $\textbf{X}^\varepsilon$ and $Y_v^\varepsilon$, $\textbf{Y}^\varepsilon$, with $v\in U$, to emphasize that the domain is now on $U_\varepsilon$, so that $X^\varepsilon _v = X_{v_\varepsilon }$, and analogously with the other variables.

### Theorem 4.2

(Cumulants of ${\mathbf {X^k} \, \textbf{Y}}$ on a graph) Let $\mathcal G = (\Lambda ,\,E)$ be any graph. Let $n\ge 1$, $V{:=}\left\{ v_1,\,\dots ,\,v_n\right\} \subseteq \Lambda ^{\textrm{in}}$ be a good set, with $v_i\ne v_j$ for all $i\ne j$. For a set of edges $\mathcal {E}\subseteq E$ and $v\in V$ denote $\mathcal {E}_v{:=}\{f\in \mathcal {E}:\,f^- = v\}\subseteq E_v$. The *n*-th joint cumulants of the fields $\big (X_v^{k_v} Y_v\big )_{v\in V}$ are given by

4.1 $$\begin{aligned} \kappa \left( X_v^{k_v} Y_v:\,v\in V\right) = (-1)^{\sum _v k_v} \sum _{\mathcal {E}\subseteq E:\, |\mathcal {E}_v|\ge k_v \, \forall v} K(\mathcal {E}) \sum _{\tau \in S_{{{\,\textrm{co}\,}}}(\mathcal {E})} {{\,\textrm{sign}\,}}(\tau ) \prod _{f\in \mathcal {E}} M\left( f,\,\tau (f)\right) \end{aligned}$$

where

$$ K(\mathcal {E}){:=}\prod _{v\in V}K(\mathcal {E}_v), \quad K(\mathcal {E}_v){:=}(-1)^{|\mathcal {E}_v|}\left( {\begin{array}{c}|\mathcal {E}_{v}|\\ k_v\end{array}}\right) , $$

$M=M_{E(V)}$, and $k_v \in \mathbb {N}$ for all $v\in V$.

### Remark 5

The reader might be wondering why we work with cumulants instead of moments in this case, which in view of Theorem 3.7, seems to only introduce complications. The reason for this is that cumulants are independent of the mean, which allows us to obtain a limiting result in the next theorem without the need of renormalizing.

Let us now turn our attention to homogeneous lattices $\textbf{L}$. Specifically, we will consider the triangular lattice $\textbf{T}$ and the hexagonal lattice $\textbf{H}$ in 2 dimensions, as well as the hypercubic lattice $\mathbb Z^d$ in *d* dimensions, for $d \ge 2$. Take any two non-parallel edges incident on the same vertex *v*, for any $v\in V$, and consider the plane spanned by these two edges. Let *p* be the number of edges incident on the same *v* which are contained in this plane, and let $\alpha \in \{0,\,\dots ,\,p - 1\}$; that is, $\alpha = 4$ for $\mathbb Z^d$, 6 for $\textbf{T}$ and 3 for $\textbf{H}$. Let $\gamma _\alpha {:=}\cos \left( 2\pi \alpha /p\right)$. This next theorem is a generalization of Cipriani et al. [6, Theorems 3.6 and 5.1.2] when $k_v=1$ for all $v\in V$. We unify their statements and proofs in one theorem.

### Theorem 4.3

(Scaling limit of the cumulants of $\mathbf {X^{k} \, Y}$) Let $n\ge 2$, $V{:=}\left\{ v_1,\,\dots ,\,v_n\right\} \subseteq U$ be such that ${{\,\textrm{dist}\,}}(V,\,\partial U)>0$, and $\textbf{L}$ the lattice $\mathbb {Z}^d$ or $\textbf{T}$. Let $\left( \left( X_v^{k_v}\right) ^\varepsilon Y_v^\varepsilon \right) _v$ be defined on $U_\varepsilon = U/\varepsilon \cap \textbf{L}$. If $v_i \ne v_j$ for all $i\ne j$, then

4.2 $$\begin{aligned} \tilde{\kappa }(v_1,\,\dots ,\,v_n)&{:=}\lim _{\varepsilon \rightarrow 0} \varepsilon ^{-dn} \kappa \left( \left( X_v^{k_v}\right) ^\varepsilon \, Y_v^\varepsilon :\,v\in V\right) \nonumber \\&= - \left[ \prod _{v\in V}C_\textbf{L}^{(k_v)}\right] \sum _{\sigma \in S_{{{\,\textrm{cycl}\,}}}(V)} \sum _{\eta :\,V\rightarrow \{\tilde{e}_1,\,\ldots ,\,\tilde{e}_d\}} \prod _{v\in V} \partial _{\eta (v)}^{(1)}\partial _{\eta (\sigma (v))}^{(2)} g_U\left( v,\, \sigma (v)\right) , \end{aligned}$$

where the constants $C_\textbf{L}^{(k_v)}$ are given by4.3with $c_{\mathbb {Z}^d} = 2$ for all $d\ge 2$, $c_{\textbf{T}} = 3$, and for any $f,\,g\in E_v$

$$ \overline{M}(f,\,g) = \nabla _{\eta ^*(f)}^{(1)}\nabla _{\eta ^*(g)}^{(2)}G_{0}(f^{-},\,g^-) $$

and

4.4 $$\begin{aligned} \overline{M}^\alpha (f,\,g) = {\left\{ \begin{array}{ll} \overline{M}(e_1,\,g) &  \text { if } f = e_{1+\alpha },\\ \overline{M}(f,\,g) &  \text { if } f \ne e_{1+\alpha }. \end{array}\right. } \end{aligned}$$

### Remark 6

The reader might have noticed that expression (4.2) can be expressed as a sum of traces of a product of matrices. More precisely,

$$ (4.2) = - \left[ \prod _{v\in V}C_\textbf{L}^{(k_v)}\right] \sum _{\sigma \in S_{{{\,\textrm{cycl}\,}}}(V)} \textrm{Tr}\left( \prod _{v\in V} G(v,\, \sigma (v))\right) . $$

with the matrix $G = (G_{i,\,j})_{i,\,j}$ as

$$ G_{i,\,j}(v,\, \sigma (v)) {:=}\partial _{e_i}^{(1)}\partial _{e_j}^{(2)} g_U\left( v,\, \sigma (v)\right) . $$

### Remark 7

As we will see in the proof, the same techniques are immediately generalizable to the hexagonal lattice; that is, for $\textbf{L}= \textbf{H}$. However, that case requires more care, since we have to account for the two types of vertices in that lattice. We believe an adaptation of the proof to that case only adds obscurity to the matter, but nonetheless it can still be done, yielding the same expression with $p=3$ and $c_{\textbf{H}} = 3/2$.

### Remark 8

After the proof of this theorem, on page 23 we provide a table with the explicit values of $C_{\textbf{L}}^{(k)}$ for $\mathbb {Z}^2$, $\textbf{T}$ and $\textbf{H}$, using the potential kernel values of the lattices (see e.g. Kenyon and Wilson [10] or Poncelet and Ruelle [17]). The reader can observe in the table that $C_{\textbf{H}}^{(2)} = 0$, which means that any cumulant involving $k_v=2$ at any *v* automatically vanishes on the hexagonal lattice.

**What about neighboring points?** A natural question that arises is whether we can relax the good set condition on the set *V* in theorems 3.7 and 4.2. The answer is yes, as we explain below.

Let $\mathcal G = (\Lambda ,\,E)$ be any graph, *T* a realization of the UST distribution, and $v\sim w\in \Lambda$. Then

$$\begin{aligned} \mathbb {P}\left( D_v = k_v,\, D_w = k_w\right) =\\ \mathbb {P}\left( D_v = k_v,\, D_w = k_w,\, \{v,\,w\}\in T\right) + \mathbb {P}\left( D_v = k_v,\, D_w = k_w,\, \{v,\,w\}\notin T\right) . \end{aligned}$$

As we saw in Remark 2, the condition $\{v,\,w\}\in T$ translates, in the fermionic language, to introducing the multiplicative factor $\zeta (\{v,\,w\})$, whereas for $\{v,\,w\}\notin T$ we need to introduce $1-\zeta (\{v,\,w\})$. In view of Theorem 4.2, we have

$$\begin{aligned} \kappa \left( X_v^{k_v}Y_v,\, X_w^{k_w}Y_w,\, \zeta (\{v,\,w\})\right) = (-1)^{k_v+k_w} \sum _{\begin{array}{c} |\mathcal {E}_v|\ge k_v-1\\ \{v,\,w\}\notin \mathcal {E}_v \end{array}} \, \sum _{\begin{array}{c} |\mathcal {E}_w|\ge k_w-1\\ \{v,\,w\}\notin \mathcal {E}_w \end{array}} (-1)^{|\mathcal {E}_v|+|\mathcal {E}_w|} \times \\ \left( {\begin{array}{c}|\mathcal {E}_v|\\ k_v-1\end{array}}\right) \left( {\begin{array}{c}|\mathcal {E}_w|\\ k_w-1\end{array}}\right) \sum _{\tau \in S_{{{\,\textrm{co}\,}}}(\mathcal {E})} {{\,\textrm{sign}\,}}(\tau ) \prod _{f\in \mathcal {E}} M\left( f,\,\tau (f)\right) . \end{aligned}$$

Equivalently,

$$\begin{aligned} \kappa \left( X_v^{k_v}Y_v,\, X_w^{k_w}Y_w,\, 1-\zeta (\{v,\,w\})\right) = (-1)^{k_v+k_w} \sum _{\begin{array}{c} |\mathcal {E}_v|\ge k_v\\ \{v,\,w\}\notin \mathcal {E}_v \end{array}}\sum _{\begin{array}{c} |\mathcal {E}_w|\ge k_w\\ \{v,\,w\}\notin \mathcal {E}_w \end{array}} (-1)^{|\mathcal {E}_v|+|\mathcal {E}_w|} \times \\ \left( {\begin{array}{c}|\mathcal {E}_v|\\ k_v\end{array}}\right) \left( {\begin{array}{c}|\mathcal {E}_w|\\ k_w\end{array}}\right) \sum _{\tau \in S_{{{\,\textrm{co}\,}}}(\mathcal {E})} {{\,\textrm{sign}\,}}(\tau ) \prod _{f\in \mathcal {E}} M\left( f,\,\tau (f)\right) . \end{aligned}$$

With these expressions we can calculate the moments that give the sought-after probabilities. This is immediately generalized to the case of an arbitrary finite number of points.

**Complete graphs** It is shown in Pemantle [16, Theorem 1.3] that, for any complete graph $K_n$ with *n* vertices, as *n* goes to infinity the degree of the UST at any vertex *v* converges in distribution to a random variable $1+\mathcal P(1)$, being $\mathcal P(1)$ a Poisson variable with parameter 1. This can also be obtained as a corollary from our Theorem 4.2 in a much shorter way, as follows:

### Theorem 4.4

(Pemantle [16, Theorem 1.3]) Let $K_n$ be a complete graph with *n* vertices, and let $V(K_n)$ be its vertex set. For any $v\in V(K_n)$ it holds that

$$ D_v \xrightarrow [n\rightarrow \infty ]{\textrm{dist}} 1 + \mathcal P(1), $$

with $\mathcal P(1)$ a Poisson random variable with parameter 1.

### Alternative simpler proof

From Theorem 4.2, for $k = 1,\,\ldots ,\,n$ we have that

$$ \mathbb {P}(D_v = k) = (-1)^k \sum _{\mathcal {E}\in E_v:\,\mathcal {E}\ge k} (-1)^{|\mathcal {E}|} \left( {\begin{array}{c}|\mathcal {E}|\\ k\end{array}}\right) \det (M)_\mathcal {E}. $$

According to Pemantle [16], the matrix *M* for a complete graph $K_n$ is given by

$$ M(e,\,f) = {\left\{ \begin{array}{ll} 2/n &  \text {if } e = f,\\ 1/n &  \text {if } e\ne f. \end{array}\right. } $$

Straightforward calculations then yield

$$ \det (M)_\mathcal {E}= \frac{1+|\mathcal {E}|}{n^{|\mathcal {E}|}} . $$

This way,

$$\begin{aligned} \mathbb {P}(D_v = k)= &   (-1)^k \sum _{\mathcal {E}\in E_v:\,\mathcal {E}\ge k} (-1)^{|\mathcal {E}|} \left( {\begin{array}{c}|\mathcal {E}|\\ k\end{array}}\right) \frac{1+|\mathcal {E}|}{n^{|\mathcal {E}|}}\\= &   (-1)^k \sum _{k'=k}^{n-1} \left( {\begin{array}{c}n-1\\ k'\end{array}}\right) (-1)^{k'} \left( {\begin{array}{c}k'\\ k\end{array}}\right) \frac{1+k'}{n^{k'}} . \end{aligned}$$

After algebraic manipulations,

$$ \mathbb {P}(D_v = k) = (1 + k) (n-1)^{-(2+k)} \left( \frac{n-1}{n}\right) ^n n \left[ n \left( {\begin{array}{c}n-1\\ k\end{array}}\right) - \left( {\begin{array}{c}n\\ 1 + k\end{array}}\right) \right] . $$

Taking the limit $n\rightarrow \infty$,

$$ \mathbb {P}(D_v = k) \xrightarrow {n\rightarrow \infty } \frac{e^{-1}}{(k-1)!}, \quad k\ge 1, $$

which exactly matches the distribution of a random variable $1+\mathcal P(1)$. $\square$

### Remark 9

As Pemantle [16, Section 5.2] mentions, this result holds for a more general set of graphs, which the author calls *Gino-regular graphs*, and the proof follows in the same way. A sequence of graphs $(\mathcal G_n)_n$ is called Gino-regular if there exists a sequence of positive integers $(D_n)_n$ such that

(i) the maximum and minimum degree of any vertex in $\mathcal G_n$ behave as $(1+ o(1)) D_n$ as $n\rightarrow \infty$, and
(ii) the maximum and minimum over vertices $x,\,y,\,z,\,x\ne y$ of $\mathcal G_n$ of the probability that a symmetric random walk on $\mathcal G_n$ started at *x* hits *y* before *z* behaves as $1/2 + o(1)$ as $n\rightarrow \infty$,

where by *o*(1) we intend a quantity that vanishes as $n\rightarrow \infty$. The set of complete graphs $(K_n)_n$ satisfy these conditions, and so do the *n*-cubes.

This type of graphs allow for an asymptotic calculation of the determinant of *M*, so that in the limit we obtain the same results as in the case of the complete graph.

## Proofs of Theorems 3.7, 4.2 and 4.3

### Proof of Theorem 3.7

The first equality is trivial from the fact that  for any random variable *X* and any measurable set *A*. Let us then prove the second equality, starting with a simple lemma.

#### Lemma 5.1

The degree-*k* fields satisfy

5.1 $$\begin{aligned} \mathbb {E}\left[ \prod _{v\in V}\delta ^{(k_v)}_v\right] = \sum _{\begin{array}{c} \eta :\,V\rightarrow 2^{E_o}\\ |\eta (v)|= k_v\; \forall v\in V \end{array}} \mathbb {P}\big (\left\{ e \in T \ \forall \, e \in \eta (V)\right\} \, \cap \, \left\{ e' \notin T \ \forall \, e'\in E(V) \setminus \eta (V)\right\} \big ), \end{aligned}$$

where $\eta (V)$ is an abuse of notation for $\cup _{v\in V}\eta (v)$.

#### Proof

This is immediate from the fact that, for any random variable *X*, any $\mathcal I\subset \mathbb {N}$, and measurable sets $A_i$ with $i\in \mathcal I$, . $\square$

Before we proceed with the proof, let us recall Proposition 4.4 from Cipriani et al. [6].

#### Proposition 5.2

Let $\mathcal G=(\Lambda ,\, E)$ be a finite graph. For all subsets of edges $S\subseteq E$

5.2 $$\begin{aligned} \mathbb {P}(T:\, S\subseteq T) = \left\langle \prod _{f\in S}\zeta (f)\right\rangle . \end{aligned}$$

#### Proof of Theorem 3.7

In view of Lemma 5.1, take any $\eta :\, V\rightarrow 2^{E(V)}$, with $|\eta (v)|= k_v$, $k_v\in \{1,\,\dots ,\,\deg _\mathcal {G}(v)\}$, for each $v\in V$. First we observe that

$$\begin{aligned} \bigcap _{v\in V}\left( \left\{ \eta (v)\subseteq T \right\} \cap \left( \bigcup _{e\in E_v\setminus \{\eta (v)\}}\{e\in T\}\right) ^c\right) = \bigcap _{v\in V}\left\{ \eta (v)\subseteq T \right\} \cap \left( \bigcup _{e\in E(V)\setminus \{\eta (V)\}}\{e\in T\}\right) ^c. \end{aligned}$$

By the inclusion–exclusion principle,

5.3 $$\begin{aligned} \mathbb {P}\left( \bigcap _{v\in V}\left( \left\{ \eta (v)\subseteq T \right\} \cap \left( \bigcup _{e\in E_v\setminus \{\eta (v)\}}\{e\in T\}\right) ^c\right) \right) \nonumber \\ \begin{aligned}&= \mathbb {P}\left( \bigcap _{v\in V}\{\eta (v)\subseteq T \}\right) - \mathbb {P}\left( \bigcap _{v\in V}\left\{ \eta (v) \subseteq T\right\} \cap \bigcup _{e\in E(V)\setminus \{\eta (V)\}}\,\{e\in T\}\right) \\&= \sum _{S \subseteq E(V) \backslash \eta (V)} (-1)^{|S|}\mathbb {P}\left( \bigcap _{v\in V}\{\eta (v) \subseteq T\}\cap (S \subseteq T) \right) , \end{aligned} \end{aligned}$$

where we sum over the probabilities that the edges of $\eta (V)$ are in the spanning tree *T* as well as those in $S\subseteq E(V) \backslash \eta (V)$. By Proposition 5.2, this becomes

5.4 $$\begin{aligned} \sum _{S \subseteq E(V) \backslash \eta (V)} (-1)^{|S|} \left\langle \prod _{\{r,\,s\} \in \eta (V)}\zeta (\{r,\,s\})\prod _{\{u,\,w\}\in S}\zeta (\{u,\,w\})\right\rangle . \end{aligned}$$

By the anticommutation relation, the sets of edges *S* such that $S \cap \eta (V) \ne \emptyset$ do not contribute to (5.4). This way,

$$\begin{aligned} \sum _{S \subseteq E(V) } \left\langle \prod _{\{r,\,s\}\in \eta (V)}\zeta (\{r,\,s\})\prod _{\{u,\,w\}\in S}(-1)^{|S|}\zeta (\{u,\,w\})\right\rangle \\ \begin{aligned}&=\left\langle \prod _{\{r,\,s\}\in \eta (V)}\zeta (\{r,\,s\})\sum _{S\subseteq E(V) }\prod _{\{u,\,w\}\in S}(-1)^{|S|}\zeta (\{u,\,w\})\right\rangle \\&=\left\langle \prod _{\{r,\,s\}\in \eta (V)}\zeta (\{r,\,s\})\prod _{\{u,\,w\}\in E(V) }\big (1-\zeta (\{u,\,w\})\big )\right\rangle . \end{aligned} \end{aligned}$$

Observing that the first product is

$$ \prod _{\{r,\,s\}\in \eta (V)}\zeta (\{r,\,s\}) = \prod _{v\in V} \prod _{e\in \eta (v)}\zeta (e) $$

and summing over all possible such $\eta$’s, we obtain the result. $\square$

### Proof of Theorem 4.2

#### Proof of Theorem 4.2

Call $Z_v^{(k_v)}{:=}X_v^{k_v}\, Y_v$. Using the same arguments as in the proof of Cipriani et al. [6, Theorem 3.5] we get

$$\begin{aligned} \kappa \left( Z_{v_1}^{(k_{v_1})},\,\dots ,\,Z_{v_n}^{(k_{v_n})}\right) \\ =\sum _{\eta } \sum _{A} (-1)^{|A|} \sum _{\pi \in \Pi (V)} \left( |\pi |-1\right) \!!\, (-1)^{|\pi |-1} \prod _{B\in \pi } \sum _{\tau \in S(E_B)} {{\,\textrm{sign}\,}}(\tau ) \prod _{f\in E_B} M\left( f,\,\tau (f)\right) , \end{aligned}$$

where the sum over $\eta$’s is over all functions $\eta :\, V \rightarrow E(V)$ with $\eta (v)\in E_v$ for all *v*, the sum over *A*’s is over the subsets of $A \subseteq {E}(V) \setminus \eta (V)$, and $E_B=E_B(\eta ,\,A)$ is the set of edges in $\eta (V) \cup A$ that intersect sites of *B*.

Notice that $|A|=|\eta (B) \cup A| - \sum _v k_v$. Therefore, the sum above only depends on $\eta$ and *A* through $\eta (B) \cup A$. We then denote $\mathcal {E}=E(\eta ,\,A) {:=}\eta (V) \cup A$ and recall $\mathcal {E}_B = \{f\in \mathcal {E}:\, \{f^-\}\cap B\ne \emptyset \}$. For $v\in V$ we will simplify notation by writing $\mathcal {E}_{v}$ rather than $\mathcal {E}_{\{v\}}$.

We notice that for a fixed $\mathcal {E}$ there are $$ \prod _{v \in V} \left( {\begin{array}{c}|\mathcal {E}_{v}|\\ k_v\end{array}}\right) $$ choices for $\eta (V)$ and *A* yielding the same $\mathcal {E}$, so the sum above can be written as

$$\begin{aligned} \kappa \left( Z_v^{(k_v)}:\, v \in V\right) = \\ (-1)^{\sum _v k_v} \sum _{\mathcal {E}:\, |\mathcal {E}_{v}| \ge k_v\; \forall v} K(\mathcal {E}) \sum _{\pi \in \Pi (V)} \left( |\pi |-1\right) \!!\, (-1)^{|\pi |-1} \prod _{B\in \pi } \sum _{\tau \in S(\mathcal {E}_B)} {{\,\textrm{sign}\,}}(\tau ) \prod _{f\in \mathcal {E}_B} M\left( f,\,\tau (f)\right) . \end{aligned}$$

The sum over partitions $\Pi (V)$ can again be treated in much the same way as Cipriani et al. [6, Theorem 3.5], yielding

$$ \kappa \left( Z_v^{(k_v)}:\, v \in V\right) = (-1)^{\sum _v k_v} \sum _{\mathcal {E}:\, |\mathcal {E}_{v}|\ge k_v \; \forall v} K(\mathcal {E}) \sum _{\tau \in S_{{{\,\textrm{co}\,}}}(\mathcal {E})} {{\,\textrm{sign}\,}}(\tau ) \prod _{f\in \mathcal {E}} M\left( f,\,\tau (f)\right) $$

as we wanted to show. $\square$

### Proof of Theorem 4.3

#### Proof of Theorem 4.3

We will give a proof that works for both $\textbf{L}= \mathbb {Z}^d$ and $\textbf{L}= \textbf{T}$ (and $\textbf{H}$ with an exception that we will mention below). The proof is divided into four steps. In Step 1, we start from the final expression obtained in Theorem 4.2 and show that it suffices to sum over only bare permutations $\tau$, instead of the bigger set of connected permutations. This is because, in the limit $\varepsilon \rightarrow 0$, only bare permutations give non zero contributions. In Step 2, we write the expression in terms of contributions of the permutations acting locally in the vicinity of a vertex and globally mapping an edge incident to one vertex to an edge which is incident to another vertex. In Step 3 we argue that, given a permutation $\tau$ on edges and an entry edge for any given point $v\in V$, only the projection of the exit edge onto the entry edge will contribute to the final expression when we take $\varepsilon \rightarrow 0$, so we can treat the former as a new edge in the direction of the entry one, weighed by its projection. Finally, in Step 4 we identify the global multiplicative constant of the cumulants.

**Step 1.**: From Theorem 4.2 we start with the expression
  $$ \kappa \left( \left( Z_v^{(k_v)}\right) ^\varepsilon :\, v \in V\right) = (-1)^{\sum _v k_v} \sum _{\mathcal {E}:\, |\mathcal {E}_{v_\varepsilon }|\ge k_v \; \forall v} K(\mathcal {E}) \sum _{\tau \in S_{{{\,\textrm{co}\,}}}(\mathcal {E})} {{\,\textrm{sign}\,}}(\tau ) \prod _{f\in \mathcal {E}} M\left( f,\,\tau (f)\right) . $$
  This step is practically identical to Step 1 in the proof of Theorem 3.6 in Cipriani et al. [6], since it does not depend on $k_v$, so we omit the whole derivation. It is obtained that, in the limit $\varepsilon \rightarrow 0$, only *bare* permutations contribute to the final result, obtaining the expression
  5.5 $$\begin{aligned} (-1)^{\sum _v k_v} \sum _{\mathcal {E}:\, |\mathcal {E}_{v}|\ge k_v \; \forall v} K(\mathcal {E}) \sum _{\tau \in S_{{{\,\textrm{bare}\,}}}(\mathcal {E})} {{\,\textrm{sign}\,}}(\tau ) \prod _{f\in \mathcal {E}} \overline{M}\left( f,\,\tau (f)\right) , \end{aligned}$$
  where we use the notation 5.6 whenever $\eta ^*(f)=e_i$ and $\eta ^*(\tau (f)) = e_j$ for some $e_i,\,e_j\in E_o$.

#### Remark 10

In the hexagonal lattice there are two types of points: those with edges at 0, $2\pi /3$ and $4\pi /3$ degrees, and those with edges at $\pi /3$, $\pi$ and $5\pi /6$ degrees. Following the proof in  Cipriani et al. [6], this step needs extra care when dealing with the hexagonal lattice, since as $\varepsilon \rightarrow 0$, $v_\varepsilon$ alternates between the two different types of points. Nevertheless, regardless of the point, the contribution will be the same and the result holds for $\textbf{H}$ as well, but we omit this technical detail.

**Fig. 4:**
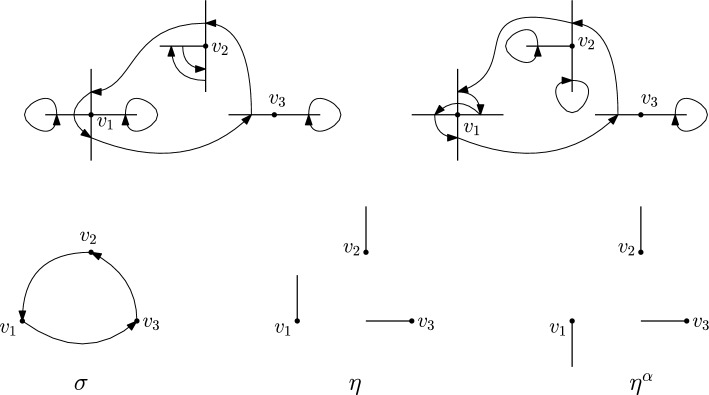
Top: two different compatible permutations in the hypercubical case in $d=2$. Bottom: their corresponding $\sigma$, $\eta$ and $\eta ^\alpha$

**Fig. 5:**
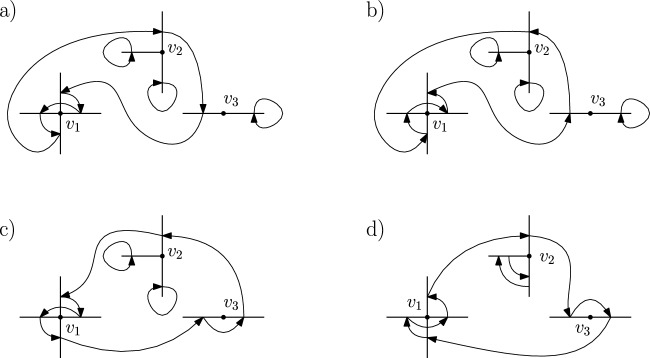
Four different permutations that are not compatible with those in Figure 4. a) Permutation that respects $\eta$ and $\eta ^\alpha$ but not $\sigma$. b) Permutation that respects $\sigma$ and $\eta ^\alpha$ but not $\eta (v_1)$. c) Permutation that respects $\sigma$ and $\eta$ but not $\eta ^\alpha (w_3)$. d) Permutation that does not respect $\sigma$, nor $\eta (v_1)$, nor $\eta ^\alpha (w_3)$

**Fig. 6:**
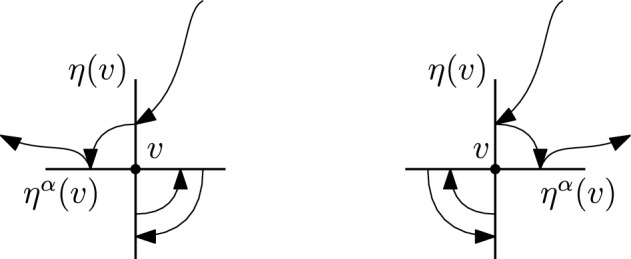
Square lattice in $d=2$. Left: a permutation $\tau$ on *v*. Right: its reflection $\rho$

**Fig. 7:**
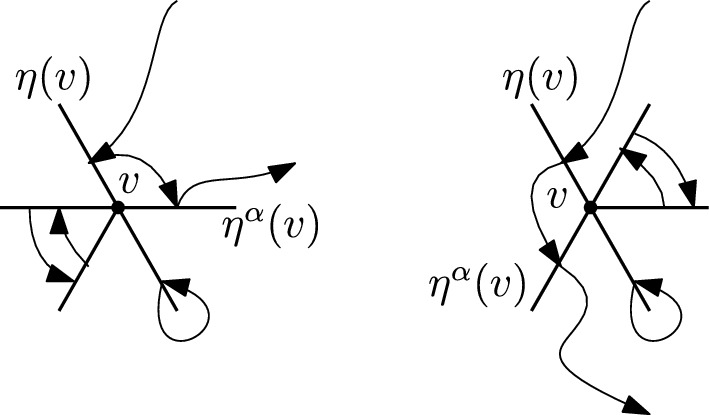
Triangular lattice in $d=2$. Left: a permutation $\tau$ on *v*. Right: its reflection $\rho$

**Fig. 8:**
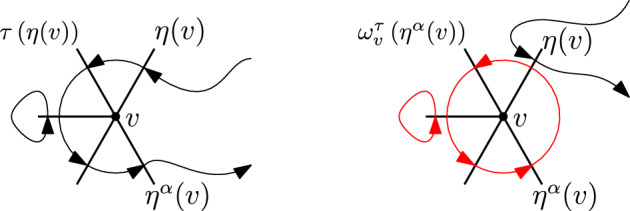
Left: a permutation $\tau$ at the point *v*. Right: the surgery applied to $\tau$, with $\omega ^\tau _v$ denoted in red

**Step 2.**: Given $\tau \in S_{{{\,\textrm{bare}\,}}}(\mathcal {E})$, fix $v\in V$, and let $\eta (v)=\eta (v,\,\tau )$ be the edge through which $\tau$ enters *v*. Let $\alpha (v) \in \{0,\,\dots ,\,p - 1\}$, where *p* is the number of edges contained in any two dimensional plane generated by any two edges incident on any $v\in V$; that is, 4 for the hypercubic lattice in *d* dimensions and 6 for the triangular lattice. We define $\eta ^\alpha (v)$ as the edge through which $\tau$ exists *v*, and $2\pi \alpha (v)/p$ denotes the angle between the entry and exit edges. Let $\gamma _\alpha (v) {:=}\cos \left( 2\pi \alpha (v)/p\right)$, so that
  $$ \langle \eta (v),\,\eta ^\alpha (v)\rangle = \gamma _\alpha (v). $$
  In the case of the hypercubic lattice the angles between entry and exit edges are multiples of $\pi /2$, hence their cosines belong to $\{-1,\,0,\,1\}$, whereas in the triangular lattice in $d=2$ angles are multiples of $\pi /3$, and their cosines belong to $\{-1,\,-1/2,\,0,\,1/2,\,1\}$.
  As stated in Section 4, any bare $\tau$ induces a permutation $\sigma \in S_{{{\,\textrm{cycl}\,}}}(V)$ on vertices. We will extract from $\tau$ a permutation $\sigma$ among vertices and a choice of edges $\eta$, and we will separate it from what $\tau$ does “locally” in the edges corresponding to a given point. Note that $\eta$, $\sigma$ and $\alpha$ determine $E_\tau (V)$ and are functions of $\tau$ (we will not write this to avoid heavy notation). With the above definitions we have that (5.5) becomes
  5.7 $$\begin{aligned}&(-1)^{\sum _v k_v} \sum _{\mathcal {E}:\, |\mathcal {E}_{v}|\ge k_v \; \forall v} \sum _{\begin{array}{c} \eta :\,V\rightarrow E(V)\\ \eta (v)\in \mathcal {E}_v\; \forall v \end{array}}\; \sum _{\sigma \in S_{{{\,\textrm{cycl}\,}}}(V)} \sum _{\alpha :\,V\rightarrow \{0,\,\ldots ,\,p-1\}} \left( \prod _{v\in V} K(\mathcal {E}_v)\overline{M}\left( \eta ^\alpha (v),\,\eta (\sigma (v))\right) \right) \times \nonumber \\&\quad \times \sum _{\tau \in S_{{{\,\textrm{bare}\,}}}(\mathcal {E};\,\eta ,\,\sigma ,\,\alpha )}{{\,\textrm{sign}\,}}(\tau ) \prod _{f\in \mathcal {E}\setminus \{\eta ^\alpha (V)\}} \overline{M}\left( f,\,\tau (f)\right) , \end{aligned}$$
  where $\eta ^\alpha (V) {:=}\{\eta ^\alpha (v):\,v\in V\}$, and $S_{{{\,\textrm{bare}\,}}}(\mathcal {E};\,\eta ,\,\sigma ,\,\alpha )$ is the set of bare permutations which now enter and exit each point *v* through the edges prescribed by $\eta$, $\sigma$ and $\alpha$. In this case we will say that $\tau$ is compatible with $(\mathcal {E};\,\eta ,\,\sigma ,\,\alpha )$. Figures 4 and 5 give examples of compatible resp. non-compatible pairs of permutations for the hypercubic lattice in $d=2$.
**Step 3.**: Define $R_{v,\,\eta ,\,\alpha }:\, \mathbb {R}^d \rightarrow \mathbb {R}^d$ to be the reflection perpendicular to the line given by $\eta (v)$, parallel to the plane generated by $\eta (v)$ and $\eta ^\alpha (v)$ (in case they are co-linear the reflection is the identity). More precisely, let us call $\mathfrak S$ the plane generated by $\eta (v)$ and $\eta ^\alpha (v)$, assuming they are not co-linear. Any edge $e\in \mathcal {E}$ can always be decomposed as
  $$ e = \mathcal P^{\mathfrak S}(e) + \mathcal P^{\mathfrak S^\perp }(e), $$
  being $\mathcal P^{\mathfrak S}$ (resp. $\mathcal P^{\mathfrak S^\perp }$) the orthogonal projection operator on $\mathfrak S$ (resp. $\mathfrak S^\perp$, that is, the orthogonal complement of $\mathfrak S$ on $\mathbb {R}^d$). In turn, this can be further decomposed as
  $$ e = \mathcal P^{\mathfrak S}(e)_{\eta (v)} + \mathcal P^{\mathfrak S}(e)_{\eta (v)^\perp } + \mathcal P^{\mathfrak S^\perp }(e), $$
  being $\mathcal P^{\mathfrak S}(e)_{\eta (v)}$ the component of $\mathcal P^{\mathfrak S}(e)$ in the direction of $\eta (v)$, and $\mathcal P^{\mathfrak S}(e)_{\eta (v)^\perp }$ its orthogonal complement. Of course, $\mathcal P^{\mathfrak S}(e)_{\eta (v)} =(e)_{\eta (v)}$, that is, the component (or projection) of *e* in the direction of $\eta (v)$. Let us rewrite this as
  $$ e = \mathcal P^{\mathfrak S}(e)_{\eta (v)^\perp } + e' $$
  for some unique $e'\in \mathbb {R}^d$. We then define $R_{v,\,\eta ,\,\alpha }:\, \mathbb {R}^d \rightarrow \mathbb {R}^d$ as
  $$ R_{v,\,\eta ,\,\alpha }(e) {:=}-\mathcal P^{\mathfrak S}(e)_{\eta (v)^\perp } + e'. $$
  We then define
  $$ \mathcal {E}' {:=}R_{v,\,\eta ,\,\alpha }(\mathcal {E}){:=}\left( \bigcup _{v' \ne v} \mathcal {E}_{v'}\right) \cup \left\{ R_{v,\,\eta ,\,\alpha }(e):\, e \in \mathcal {E}_v\right\} $$
  and, for $\tau \in S_{{{\,\textrm{bare}\,}}}(\mathcal {E})$, define $\rho \in S_{{{\,\textrm{bare}\,}}}(\mathcal {E}')$ as
  $$\begin{aligned} \rho (e) = {\left\{ \begin{array}{ll} \tau (e) &  \text { if } e \in \cup _{v' \ne v}\mathcal {E}_{v'},\\ \tau (\eta ^\alpha (v)) & \text { if } e = R_{v,\,\eta ,\,\alpha }(\eta ^\alpha (v)),\\ R_{v,\,\eta ,\,\alpha }(\tau (e')) & \text { if } e=R_{v,\,\eta ,\,\alpha }(e') \text { for some } e'\in \mathcal {E}_v\setminus \{\eta ^\alpha (v)\} . \end{array}\right. } \end{aligned}$$
  See Figure 6 for an example of the reflected permutation $\rho$ in the square lattice, and Figure 7 for the triangular lattice. We can then see that $K(\mathcal {E})=K(\mathcal {E}')$ and ${{\,\textrm{sign}\,}}(\tau )={{\,\textrm{sign}\,}}(\rho )$. Furthermore, with simple calculations of inner products we have
  5.8 $$\begin{aligned}  &   \overline{M}\left( \eta ^\alpha (v),\,\eta (\sigma (v))\right) + \overline{M}\left( R_{v,\eta }(\eta ^\alpha (v)),\,\eta (\sigma (v))\right) \nonumber \\  &   \quad = 2\cos \left( \frac{2\pi \alpha (v)}{p}\right) \,\overline{M}(\eta (v),\,\eta (\sigma (v))). \end{aligned}$$
  Observe that these cancellations happen in the hypercubic, triangular and hexagonal lattices due to their high symmetries.
  With 5.8 in mind, Equation (5.7) becomes
  5.9 $$\begin{aligned} (-1)^{\sum _v k_v} \sum _{\mathcal {E}:\, |\mathcal {E}_v|\ge k_v \, \forall v} \sum _{\begin{array}{c} \eta :\,V\rightarrow E(V)\\ \eta (v)\in \mathcal {E}_v\; \forall v \end{array}}\;\sum _{\sigma \in S_{{{\,\textrm{cycl}\,}}}(V)} \sum _{\alpha :\,V\rightarrow \{0,\,\ldots ,\,p-1\}} \sum _{\tau \in S_{{{\,\textrm{bare}\,}}}(\mathcal {E};\,\eta ,\,\sigma ,\,\alpha )} {{\,\textrm{sign}\,}}(\tau ) \times \nonumber \\ \times \prod _{f\in \mathcal {E}\setminus \eta ^{\alpha }(V)} \overline{M}\left( f,\,\tau (f)\right) \prod _{v\in V}K(\mathcal {E}_v) \gamma _\alpha (v) \underbrace{\prod _{v\in V} \partial ^{(1)}_{\eta (v)}\partial ^{(2)}_{\eta (\sigma (v))} g_{U}\left( \eta (v),\,\eta (\sigma (v))\right) }_{(\star )}. \end{aligned}$$
  The factor $(\star )$, which accounts for the interactions between different points, only depends on the entry directions given by $\eta$, not on the exit directions $\eta ^{\alpha }$. This is the key cancellation to obtain expressions of the form (4.2), up to constant.
  We rewrite expression (5.9) as
  5.10 $$\begin{aligned} \sum _{\begin{array}{c} \eta :\,V\rightarrow E(V)\\ \eta (v)\in E_{v}\; \forall v \end{array}}\; \sum _{\sigma \in S_{{{\,\textrm{cycl}\,}}}(V)} \prod _{v\in V} \partial ^{(1)}_{\eta (v)}\partial ^{(2)}_{\eta (\sigma (v))} g_{U}\left( \eta (v),\,\eta (\sigma (v))\right) \times \nonumber \\ \times \prod _{v\in V}\underbrace{(-1)^{k_v} \sum _{\begin{array}{c} \mathcal {E}_v:\, \mathcal {E}_v\ni \eta (v)\\ |\mathcal {E}_v|\ge k_v \end{array}} K(\mathcal {E}_v) \sum _{\alpha =0}^{p-1} \gamma _\alpha (v) \sum _{\tau \in S_{{{\,\textrm{bare}\,}}}(\mathcal {E};\,\eta ,\,\sigma ,\,\alpha )}{{\,\textrm{sign}\,}}(\tau ) \prod _{f\in \mathcal {E}_v\setminus \{\eta ^\alpha (v)\}}\overline{M}\left( f,\,\tau (f)\right) }_{(\star \star )}. \end{aligned}$$
  Remark that if $\eta ^\alpha (v) \not \in {\mathcal {E}}$, the set $S_{{{\,\textrm{bare}\,}}}(\mathcal {E};\,\eta ,\,\sigma ,\,\alpha )$ is empty, and therefore not contributing to the sum.
  Notice that all entries of the type $\overline{M}(e,\,\tau (e))$ in $(\star \star )$ are discrete double gradients of the Green function of the full lattice $\textbf{L}$ (see Equation (5.6)). In the following we will prove that $(\star \star )$ does not depend on the choice of $\eta$ nor $\sigma$. The value of the term $(\star \star )$ will give the constants $C_\textbf{L}^{(k_v)}$ (up to an overall minus sign).
**Step 4.**: Using $\sigma$, $\eta$ and $\alpha$, we have been able to isolate in (5.10) an expression that depends only on permutations of vertices. To complete the proof we will perform a “surgery” to better understand expression (5.10). This surgery aims at decoupling the local behavior of $\tau$ at a vertex versus the jumps of $\tau$ between different vertices.
  To do this, given $\eta :\, V \rightarrow E(V)$, $\alpha : V \rightarrow \{0,\,\dots ,\,p-1\}$, $\mathcal {E} \subseteq E(V)$ with $\eta (v),\, \eta ^\alpha (v) \in \mathcal {E}_v$, and $\tau \in S_{{{\,\textrm{bare}\,}}}(\mathcal {E};\,\eta ,\,\sigma ,\,\alpha )$, we define $\omega _v^\tau (\mathcal {E}_v \setminus \{\eta (v)\})$ and $\tau \setminus \omega ^\tau _v ( (\mathcal {E} \setminus \mathcal {E}_v )\cup \{\eta (v)\})$ as
  5.11 $$\begin{aligned} \omega ^\tau _v(f){:=}{\left\{ \begin{array}{ll} \tau (f)& \text {if }f\ne \eta ^\alpha (v)\\ \tau (\eta (v))& \text {if }f= \eta ^{\alpha }(v),\,\alpha (v) \ne 0 \end{array}\right. },\quad f\in \mathcal {E}_v\setminus \{\eta (v)\} \end{aligned}$$
  and
  $$\begin{aligned} \tau \setminus \omega ^\tau _v(f){:=}{\left\{ \begin{array}{ll} \tau (f)& \text {if }f\notin \mathcal {E}_v\\ \eta (\sigma (v))& \text {if }f= \eta (v) \end{array}\right. },\quad f\in (\mathcal {E}\setminus \mathcal {E}_v) \cup \{\eta (v)\}. \end{aligned}$$
  In words, $\omega ^\tau _v$ is the permutation induced by $\tau$ on $\mathcal {E}_v\setminus \{\eta (v)\}$ by identifying the entry and the exit edges. On the other hand, $\tau \setminus \omega ^\tau _v(f)$ follows $\tau$ globally until it reaches the edges incident to $v_\varepsilon$, from where it departs reaching the edges of the next point. An example of $\omega _v^{\tau }$ for the triangular lattice can be found in Figure 8.
  In the following we state two technical lemmas the we need to complete the proof of the theorem. These are identical to Lemmas 4.8 and 4.9/5.3 in  Cipriani et al. [6], so we omit their proofs.

#### Lemma 5.3

Let $\mathcal {E}\subseteq E(V)$, $\eta :\,V\rightarrow E(V)$ such that $\eta (v)\in \mathcal {E}_v$ for all $v\in V$, $\sigma \in S_{{{\,\textrm{cycl}\,}}}(V)$, $\alpha :\, V\rightarrow \{0,\,\ldots ,\,p-1\}$ and let $\tau$ be compatible with $(\mathcal {E};\,\eta ,\,\sigma ,\,\alpha )$. For every $v\in V$ there is a bijection between $S(\mathcal {E}_v\!\setminus \!\{\eta (v)\})$ and $\{\omega ^\tau _v:\,\tau \text { { compatible with }} (\mathcal {E};\,\eta ,\,\sigma ,\,\alpha )\}$.

#### Lemma 5.4

(Surgery of $\tau$) Fix $v\in V$ and $\mathcal {E}$, $\eta$, $\sigma$, $\alpha$ as above. Let $\tau$ be compatible with $\mathcal {E}$, $\eta$, $\sigma$ and $\alpha$. Then5.12Furthermore,

$$ \prod _{f\in \mathcal {E}_{v}\setminus \{\eta ^\alpha (v)\}} \overline{M}\left( f,\,\tau (f)\right) = \frac{\overline{M}\left( \eta (v),\,\omega ^\tau _v(\eta ^\alpha (v))\right) }{\overline{M}\left( \eta ^\alpha (v),\,\omega ^\tau _v(\eta ^\alpha (v))\right) } \prod _{f\in \mathcal {E}_{v}\setminus \{\eta (v)\}} \overline{M}\left( f,\,\tau (f)\right) . $$

Equivalently, we can write that

5.13 $$\begin{aligned} \prod _{f\in \mathcal {E}_v\setminus \{\eta ^\alpha (v)\}} \overline{M}\left( f,\,\tau (f)\right) = \prod _{f\in \mathcal {E}_v\setminus \{\eta (v)\}} \overline{M}^{\alpha }\left( f,\,\omega ^\tau _v(f)\right) , \end{aligned}$$

where for any $g\in \mathcal {E}_v$

5.14 $$\begin{aligned} \overline{M}^{\alpha }\left( f,\,g\right) :={\left\{ \begin{array}{ll} \overline{M}(\eta (v),\,g) &  \text { if } f=\eta ^{\alpha }(v), \\ \overline{M}(f,\,g) &  \text { if } f\ne \eta ^{\alpha }(v). \end{array}\right. } \end{aligned}$$

Remark that the matrix $\overline{M}^{\alpha }$ is not symmetric anymore. We will now use these lemmas to rewrite (5.10) in a more compact form. Using (5.12) recursively, we getNote that the permutation $(((\tau \setminus \omega ^\tau _{v_1})\setminus \omega ^\tau _{v_2})\setminus \ldots )\setminus \omega ^\tau _{v_n}$ equals the permutation

$$ (\eta (v_1),\,\eta (\sigma (v_1)),\,\eta (\sigma (\sigma (v_1))),\,\ldots ,\,\eta (\sigma ^{n-1}(v_1))) $$

and, as such, it constitutes a cyclic permutation on *n* edges in $\mathcal {E}$, so that

$$ {{\,\textrm{sign}\,}}((((\tau \setminus \omega ^\tau _{v_1})\setminus \omega ^\tau _{v_2})\setminus \ldots )\setminus \omega ^\tau _{v_n}) = (-1)^{n-1}. $$

With this in mind, applying (5.13) at every *v* we can rewrite $\prod _{v\in V}(\star \star )$ asRecall that, given $\alpha (v)$, $\omega _v^\tau (\eta ^\alpha (v))=\tau (\eta (v))$, which means that now the dependence on $\tau$ is only through $\omega _v^\tau$ and $\alpha (v)$. This, together with Lemma 5.3, allows us to obtain5.15At this point, we note that the expression above does not depend on $\sigma$ or $\eta$ anymore, and only depends on *v* through $k_v$. In fact, as $\omega _v(f)^-=f^{-}=v$, we have that $\overline{M}\left( f,\,\omega _v(f)\right)$ is a constant by definition (see (5.6)). Therefore, without loss of generality, we can take $v=o$, $\eta (v)=e_1$ to get that (5.15) is equal to minus the product over *v* ofUsing the definition of determinant of a matrix, after applying the sum on $\alpha \in \{0,\,\ldots ,\,p-1\}$ the first term in the square brackets above is equal to $\det \left( \overline{M}\right) _{\mathcal {E}_o\setminus \{e_1\}}$, while for $\alpha \ne 0$ the second one yields , with $\overline{M}^\alpha$ as in (4.4). Summing these contributions we obtain the cumulants

$$\begin{aligned} - \left[ \prod _{v\in V}C_\textbf{L}^{(k_v)}\right] \left( \frac{1}{c_{\textbf{L}}}\right) ^n\sum _{\sigma \in S_{{{\,\textrm{cycl}\,}}}(V)} \sum _{\eta :\,V\rightarrow E_o} \prod _{v\in V} \partial _{\eta (v)}^{(1)}\partial _{\eta (\sigma (v))}^{(2)} g_U\left( v,\, \sigma (v)\right) = \\ - \left[ \prod _{v\in V}C_\textbf{L}^{(k_v)}\right] \sum _{\sigma \in S_{{{\,\textrm{cycl}\,}}}(V)} \sum _{\eta :\,V\rightarrow \{\tilde{e}_1,\,\ldots ,\,\tilde{e}_d\}} \prod _{v\in V} \partial _{\eta (v)}^{(1)}\partial _{\eta (\sigma (v))}^{(2)} g_U\left( v,\, \sigma (v)\right) , \end{aligned}$$

where the last change of coordinates is identical to that of  Cipriani et al. [6], beingwith $c_{\mathbb {Z}^d} = 2$ for all $d \ge 2$, and $c_{\textbf{T}} = 3$. $\square$

#### Remark 11

We highlight once again that, with the technical exception of Step 1, all the other steps follow in much the same way for $\textbf{H}$, in which case $p=3$, and the value of $c_{\textbf{H}}$ can also be calculated, obtaining $c_{\textbf{H}} = 3/2$.

We can now plug in the potential kernel values of the lattices in two dimensions (see e.g. Kenyon and Wilson [10] or Poncelet and Ruelle [17]), obtaining the following values of $C_{\textbf{L}}^{(k_v)}$:

$$\begin{aligned}&C_{\mathbb {Z}^2}^{(1)} = \frac{8}{\pi } - \frac{16}{\pi ^2} \approx 0.9253 \\&C_{\mathbb {Z}^2}^{(2)} = 18 - \frac{72}{\pi } + \frac{96}{\pi ^2} \approx 4.8085\\&C_{\mathbb {Z}^2}^{(3)} = 2 + \frac{16}{\pi } \approx 7.0930 \\&C_{\mathbb {Z}^2}^{(4)} = -2\\&C_{\textbf{T}}^{(1)} = -\frac{25}{6} - \frac{5\sqrt{3}}{2\pi } + \frac{297}{\pi ^2} - \frac{594\sqrt{3}}{\pi ^3} + \frac{972}{\pi ^4} \approx 1.3443\\&C_{\textbf{T}}^{(2)} = -\frac{35}{8} + \frac{611\sqrt{3}}{4\pi } - \frac{4077}{2\pi ^2} + \frac{3159\sqrt{3}}{\pi ^3} - \frac{4860}{\pi ^4} \approx -0.1296\\&C_{\textbf{T}}^{(3)} = \frac{239}{4} - \frac{537\sqrt{3}}{\pi } + \frac{5031}{\pi ^2} - \frac{6696\sqrt{3}}{\pi ^3} + \frac{9720}{\pi ^4} \approx -0.8286\\&C_{\textbf{T}}^{(4)} = -\frac{599}{6} + \frac{1433\sqrt{3}}{2\pi } - \frac{5832}{\pi ^2} + \frac{7074\sqrt{3}}{\pi ^3} - \frac{9720}{\pi ^4} \approx -0.3339\\&C_{\textbf{T}}^{(5)} = \frac{247}{4} - \frac{841\sqrt{3}}{2\pi } + \frac{3240}{\pi ^2} - \frac{3726\sqrt{3}}{\pi ^3} + \frac{4860}{\pi ^4} \approx -0.0497\\&C_{\textbf{T}}^{(6)} = -\frac{105}{8} + \frac{363\sqrt{3}}{4\pi } - \frac{1395}{2\pi ^2} + \frac{783\sqrt{3}}{\pi ^3} - \frac{972}{\pi ^4} \approx -0.0026\\&C_{\textbf{H}}^{(1)} = \frac{3}{4}\\&C_{\textbf{H}}^{(2)} = 0\\&C_{\textbf{H}}^{(3)} = -\frac{3}{4} \end{aligned}$$

## Data Availability

We do not analyse or generate any datasets, because our work proceeds within a theoretical and mathematical approach.
